# The Importance of Magnesium in Clinical Healthcare

**DOI:** 10.1155/2017/4179326

**Published:** 2017-09-28

**Authors:** Gerry K. Schwalfenberg, Stephen J. Genuis

**Affiliations:** ^1^Department of Family Medicine, University of Alberta, No. 301, 9509-156 Street, Edmonton, AB, Canada T5P 4J5; ^2^Faculty of Medicine, University of Alberta, 2935-66 Street, Edmonton, AB, Canada T6K 4C1; ^3^University of Calgary, Calgary, AB, Canada

## Abstract

The scientific literature provides extensive evidence of widespread magnesium deficiency and the potential need for magnesium repletion in diverse medical conditions. Magnesium is an essential element required as a cofactor for over 300 enzymatic reactions and is thus necessary for the biochemical functioning of numerous metabolic pathways. Inadequate magnesium status may impair biochemical processes dependent on sufficiency of this element. Emerging evidence confirms that nearly two-thirds of the population in the western world is not achieving the recommended daily allowance for magnesium, a deficiency problem contributing to various health conditions. This review assesses available medical and scientific literature on health issues related to magnesium. A traditional integrated review format was utilized for this study. Level I evidence supports the use of magnesium in the prevention and treatment of many common health conditions including migraine headache, metabolic syndrome, diabetes, hyperlipidemia, asthma, premenstrual syndrome, preeclampsia, and various cardiac arrhythmias. Magnesium may also be considered for prevention of renal calculi and cataract formation, as an adjunct or treatment for depression, and as a therapeutic intervention for many other health-related disorders. In clinical practice, optimizing magnesium status through diet and supplementation appears to be a safe, useful, and well-documented therapy for several medical conditions.

## 1. Introduction

According to the medical literature, we are currently experiencing two concomitant phenomena: (i) a “neglected epidemic of chronic disease” [1] and (ii) a widespread deficiency of selected nutrients [2–4]. While the importance of magnesium (Mg) and other required elements for the human organism is often well discussed in educational programs for trainees in physiology, nutrition, and dietetics, the importance of acquiring the tools necessary for credible assessment and practical management of nutrient compromise in clinical care can sometimes be lost in the education process for physicians [5]. Accordingly, recognition and assessment for biochemical deficiencies in day-to-day medical practice are sometimes overlooked. This review is specifically designed for medical practitioners as they revisit the important role of Mg in a clinically relevant way.

Magnesium is the fourth most common mineral in the human body after calcium, sodium, and potassium and is the second most common intracellular cation after potassium. Within the frame of a 70 kg individual, there is an average of 25 grams of Mg in reserve with 53% in bone, 27% in muscle, 19% in soft tissues, and less than 1% in the serum. Although serum Mg concentration (SMC) is tightly controlled with a normal serum value of 75–95 mmol/L, some research would indicate that serum levels less than 85 mmol/l should be considered deficient [6, 7].

Magnesium is involved as a cofactor in more than 300 enzyme systems and is required for such fundamental processes as energy production and nucleic acid synthesis (Table 1). Intracellular Mg stores are found in high concentration in mitochondria [8], where this element plays a pivotal role in the synthesis of ATP (adenosine triphosphate) from ADP (adenosine diphosphate) and inorganic phosphate [9]. In addition, Mg is bound to ATP in order to yield the bioactive form of ATP (Mg-ATP) and it is estimated that 3571 human proteins potentially bind to Mg+2 [10]. The biologic half-life of Mg in the body is about 1000 hours (42 days) [11].

*Objective*. Nutritional deficiency is an area of increasing importance in the field of medical science and has been found to be an important determinant of the widespread epidemic of chronic disease. As instruction in nutrition and food science has not been a focus within medical education [5], many physicians and other health providers are not sufficiently apprised of the important role of various essential minerals in the metabolic functioning and homeostasis of the human body. As Mg deficiency is a common and widespread cause of many everyday physical and mental health problems, this paper is designed to support clinical health professionals as they endeavor to assist patients presenting with health challenges.

## 2. Methods

This review of the role of Mg in clinical practice was prepared by assessing available medical and scientific literature from MEDLINE and PubMed, as well as by reviewing several books and conference proceedings. Terms searched included Mg and various conditions purported to be associated with this required mineral including osteoporosis, asthma, diabetes, eclampsia, cardiovascular disease, and cognitive function. Relevant references found in these publications were also searched in order to glean pertinent information. A primary observation, however, was that limited scientific literature is available on the issue of Mg insufficiency as it relates to health outcomes. The level of evidence (LOE) according to the Evidence-Based Medicine Toolkit used by the American Family Physician is provided in relation to the findings regarding each clinical condition.

A traditional integrated review format was chosen for this paper [12]. This type of publication approach seemed apposite when endeavoring to incorporate and synthesize extensive literature in a field with limited primary study, while at the same time endeavoring to provide a clinically useful overview of scientific information to clinicians and public health professionals. By collating information gleaned from the literature, we endeavor to provide information of clinical relevance regarding Mg including testing, required intake, correlation with disease states, and recommended interventions.

## 3. Results

### 3.1. Laboratory Assessment of Magnesium Status

When discussing levels of Mg in the studies, it is important to realize that there are several methods of measuring Mg as outlined in Table 2. The most commonly used test is the total serum magnesium concentration (SMC), but this laboratory marker has limited clinical benefit as it does not accurately reflect intracellular or total body Mg status [14]. In other words, as (i) less than one percent of total body Mg is found in the serum and (ii) the body endeavors to always maintain a normal level of serum Mg, an individual may be profoundly deficient in total body or intracellular Mg required for various cellular biochemical processes yet have a SMC within normal range. Serum ionized magnesium levels are significantly lower than SMC in diabetics [20] and patients with mild-to-moderate Alzheimer's disease [21]. Serum ionized Mg, tissue Mg, NMR for free Mg, and Mg retention tests may be more reflective of Mg adequacy but these investigations are not readily available to most clinicians.

### 3.2. Magnesium Deficiency

It is estimated that between 56 and 68% [22, 23] of Americans do not obtain enough magnesium in their diet on a daily basis to meet the recommended daily allowance (RDA) as outlined in Table 3. Widespread Mg intake is occurring for a number of reasons listed as follows:

- There are diminished levels of Mg in many processed foods and some nonorganic foods [24]. Most foods in grocery stores are processed.
- Common staples such as meat (18–29 mg/100 gm), sugar (0 mg/100 gm), and white flour (20–25 mg/100 gm) contribute less than 20% of the daily requirements of Mg.
- Cooking and boiling of produce result in a significant decline of the food's Mg content [25].
- Reduced gastrointestinal absorption of Mg occurs in the face of vitamin D deficiency, a common problem in western cultures [26].
- Medications in common usage (e.g., some antibiotics, antacids, and hypertensive drugs) diminish absorption of Mg. See Table 8 [6, 27].
- Some commonly used pesticides have the propensity to chelate minerals [28], potentially decreasing the content of Mg in soil and some crops [29].
- There is excess excretion of Mg with alcohol use and the presence of type 1 or type 2 diabetes [30].
- Smoking cigarettes reduces plasma Mg concentration [31].
- Evidence demonstrates increasing soil depletion of certain essential nutrients as a result of fertilization techniques not providing the spectrum of required minerals [32].
- There has been the expansion of monoculture agricultural techniques that have a tendency to consume and deplete specific nutrients.
- Magnesium absorption is reduced with aging by as much as 30%.

**Figure 1:**
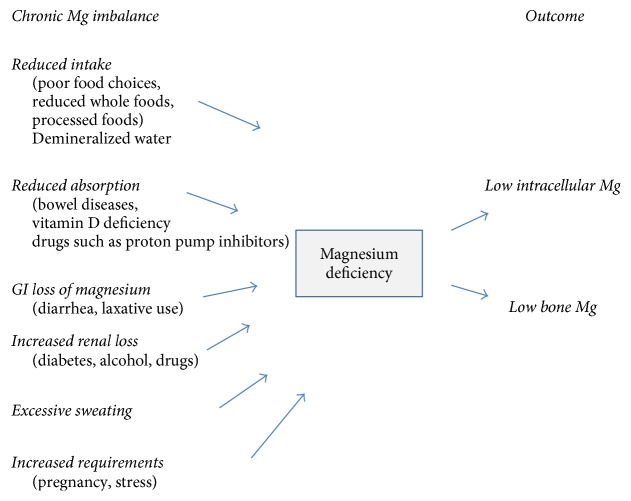
Etiology of chronic magnesium deficiency [7].

Chronic low intake of Mg may be the main reason for a total body Mg deficit; there are, however, many other factors that influence total body Mg sufficiency. A graphic representation of common pathways to chronic latent Mg deficiency is provided in Figure 1.

Chronic latent Mg deficiency is an important underlying pathology in many clinical conditions [33]. It is estimated that at least 42% of young adults have an ongoing primary Mg deficiency [34]. Clinical symptoms and signs of Mg deficiency are listed in Table 4 and it is increasingly recognized that myriad clinical presentations may reflect such a deficiency state.

### 3.3. Clinical Outcomes Related to Magnesium Deficiency

As mentioned, there are various pathways to Mg insufficiency (Table 5). Ongoing latent inadequacy of a nutrient required for hundreds of innate biochemical and physiological processes inevitably results in metabolic dysregulation with ensuing clinical manifestations. Various clinical conditions resulting from the consequent metabolic disruption of Mg deficiency will be discussed along with the associated level of evidence and supporting references.

#### 3.3.1. Magnesium and Asthma (Level of Evidence (LOE) = B)

Although mechanisms are not completely understood at this time, it appears that Mg insufficiency may be related to severe bronchospasm in some vulnerable individuals. Magnesium sulfate (MgS) given as 2 grams intravenously (IV) has shown benefit and a trend for greater improvement in the symptom of breathlessness in severe acute asthma exacerbation in a number of studies [35, 36]. A recent study showed some evidence that concomitant administration of intravenous MgS combined with standard approaches diminished the need for hospitalization in acute severe asthmatic patients compared with regular treatments alone; use of concomitant MgS by nebuliser, however, did not improve outcomes and proved no better than placebo [37].

#### 3.3.2. Magnesium, Vitamin D, Rickets, and Osteoporosis (LOE = B)

Magnesium is required for conversion of vitamin D into its active form which, in turn, supports calcium absorption and metabolism, as well as normal parathyroid hormone function [38]. Vitamin D sufficiency may then increase absorption of calcium and Mg by as much as 300% [26]. Rickets with hypomagnesaemia will not respond to massive doses of vitamin D (referred to as “vitamin D-resistant Rickets”). It is thus suggested that serum Mg levels be drawn in patients with Rickets and that Mg supplementation be considered in all such cases [39]. However, concern has been expressed that excessive doses of vitamin D may enhance urinary Mg excretion and thus result in reduced Mg retention [40].

Higher Mg intake has been associated with higher BMD in elderly white men and women [41]. In fact, oral Mg supplementation has been shown to suppress bone turnover in postmenopausal women and young adult males [42, 43]. A two-year study of individuals supplemented with Mg hydroxide resulted in less fractures and a significant increase in bone density [44]. Both excessively high and low Mg levels, however, appear to be detrimental to bone health [45]. As a result, an increase in wrist fractures was seen in the Women's Health Initiative Study in those with the highest Mg levels [46].

#### 3.3.3. Magnesium and Muscle Cramps: (LOE = A, B)

In a recent review analyzing the use of Mg supplementation for muscle cramps, the results demonstrated a trend towards benefit but the findings did not reach clinical significance [47]. A Cochrane database review looking at supplementing 360 mg/day (120 mg in the morning and 240 mg in the evening) of Mg in pregnancy, however, demonstrated efficacy with regard to ameliorating problems with muscle cramps [48]. On the other hand, a recent double-blind placebo controlled trial using the same dose resulted in no significant benefit [49]. Although the use of Mg is safe for muscle cramps, solid evidence is still lacking.

One explanation for this may be that deficiencies of other elemental nutrients including calcium and potassium have also been implicated in muscle cramps and spasms. It may be that Mg is potentially helpful in situations of Mg deficiency but is not of use if the problem is related to deficiency of another nutrient. With the confounder of multiple potential etiologies for a single presentation and no easy means to verify tissue Mg levels, it will be difficult to conclusively prove a direct link at this time.

#### 3.3.4. Magnesium and Pregnancy (LOE = A, B)

As Mg deficiency is a common event in pregnancy [50], consequences of gestational deficiency are beginning to be observed. Preliminary evidence suggests that Mg deficiency is a determinant of pregnancy outcomes as well as long-term health of the offspring [51]. Oral Mg supplementation given before the 25th week of gestation compared with placebo, for example, was associated with a lower frequency of preterm births, low birth weight infants, and fewer small for gestational age newborns [52]. One study showed that Mg supplementation in pregnancy was associated with lower mean arterial pressure in women along with higher birth weight infants and fewer days spent in the neonatal intensive care unit [53].

In the later stages of pregnancy, Mg has long been a treatment for preeclampsia/eclampsia with IV (LOE = A) MgS proving to have superior outcomes compared to diazepam or phenytoin in preventing seizures, reducing vascular resistance, and improving cardiac output. There is a 52% lower risk of developing recurrent seizures with MgS treatment. Treatment of preeclampsia with MgS reduced the rate of eclampsia by 50% [54]. Furthermore, there is preliminary evidence that fetal hypomagnesemia may be associated with metabolic syndrome later in life [50].

#### 3.3.5. Magnesium and Migraine Headaches (LOE = A)

A Cochrane review grades Mg as one of the strongly recommended treatments for migraine headaches [55]. Oral Mg supplementation has been shown to reduce the frequency, duration, and intensity of migraines by 41% compared to placebo at 15.8% [56]. Magnesium sulfate (1 gram IV) may be useful in migraine patients with aura (37% responded with less pain) but not in common migraine [57]. After a 3-month treatment period with oral Mg citrate at 600 mg/day for migraine without aura, a recent study showed significant improvement in attack frequency, severity, and P1 amplitude in visual evoked potential examination [58].

#### 3.3.6. Magnesium, Metabolic Syndrome, Diabetes, and Prevention of Diabetic Complications (LOE = A, B)

As Mg levels are generally lower in people who have metabolic syndrome [59], a diet rich in this essential element may be important in preventing metabolic syndrome. A dietary intervention study (*n* = 234) with metabolic syndrome had the Mg intake and insulin resistance estimated by the homeostasis model assessment (HOMA-IR) four times over one year. The average intake of Mg was 287 ± 93 mg/day (mean ± standard deviation) at baseline. The highest quartile of Mg intake had a 71% reduction of developing an elevated HOMA-IR compared to the lowest quartile [60]. A prospective study looking at Mg intake and incidence of diabetes, systemic inflammation, and insulin resistance in young American adults followed up for 20 years (*n* = 4497) showed a significant inverse relationship with Mg intake and hs-CRP, IL-6, fibrinogen, and HOMA-IR. There was a 47% reduced incidence of diabetes in the highest quartile of Mg intake [61]. A meta-analysis study consisting of 15 studies in the Cohorts for Heart and Aging Research in Genomic Epidemiology (CHARGE) (*n* = 52,6840) showed that Mg intake was inversely associated with fasting glucose and insulin (*p* < 0.0001) [62]. A recent meta-analysis of double-blind randomized controlled trials supplementing Mg (12 in diabetics and 6 in those with high risk of diabetes) showed improved glucose parameters in diabetics and improved insulin-sensitivity in pre-diabetics [63].

The most commonly detected electrolyte abnormality in ambulatory diabetic patients is hypomagnesia and Mg intake is inversely associated with incidence of type 2 diabetes. A recent study of diabetics (*n* = 210) showed that 88.6% had Mg intake less than the RDA and 37.1% had measurable hypomagnesia [64]. In this study, higher Mg intake was associated with an increase in HDL. Furthermore, there was an inverse relationship with Mg intake and triglycerides, waste circumference, body fat%, and BMI. All parameters were markedly improved in the quartile that had the highest intake of Mg [65]. In one research study, a 100 mg/day increase in Mg intake (by either diet or supplementation) resulted in a 15% reduction in incidence in diabetes [66]. The Nurses' Health Study and the Health Professionals Follow-Up Study have also shown an inverse relationship between Mg intake and type 2 diabetes mellitus (T2DM). The highest intake of Mg compared to the lowest one had a 36% relative risk reduction for T2DM [67].

Another study found that long-term Mg supplementation improves outcomes in neuropathy in type 1 diabetics [68]. 12% of patients supplemented with 300 mg/day for 5 years had progression of peripheral neuropathy compared to 61% who were not supplemented (placebo), representing a 500% reduction [68]. In T2DM, lower Mg levels are associated with more rapid decline in renal function [69]. Depression is common in patients with diabetes and often correlates with low Mg. Treatment with Mg chloride has been shown to be as effective for depressed mood in patients with T2DM as 50 mg of imipramine [70].

#### 3.3.7. Magnesium and Depression (LOE = B)

Magnesium sulfate has been successfully used in agitated depression as far back as 1921 [71]. In fact, rapid recovery of depression has been reported with the use of Mg glycinate or Mg taurinate [72]. There is an inverse correlation in adults between Mg intake and psychiatric states such as anxiety and depression [73]. Magnesium is required as a coenzyme to convert tryptophan to serotonin, a neurotransmitter recognized as a major determinant of mental health and mood. A systematic review suggests that Mg supplementation may prevent depression and may be useful as adjuvant therapy [74]. Both sertraline given at 150 mg/day for 4 weeks and amitriptyline given at 75 mg/day for 4 weeks have been shown to increase Mg concentration in erythrocytes. It has been suggested that this may be a possible biochemical mechanism for the effectiveness of these drugs in some patients [75].

Another intriguing mechanism that relates to Mg and mental health involves N-methyl-D-aspartic acid (NMDA). Recent research on ketamine as an NMDA receptor antagonist has shown rapid antidepressant effect and is being suggested in some circles for treatment-resistant depression. Magnesium is a natural NMDA receptor antagonist [76] and may serve as a natural antidepressant.

#### 3.3.8. Magnesium, Sleep, and Restless Leg Syndrome (LOE = B)

It is estimated that 50% of older adults have insomnia. Magnesium is a natural NMDA antagonist and a GABA agonist, both biochemical actions which have a relaxant effect and facilitate sleep [77]. Supplementation of 500 mg of Mg has been associated with significant improvement in the insomnia severity index, sleep time, sleep efficiency, sleep onset latency, serum cortisol concentration, serum renin, and melatonin [78].

In an open clinical trial and polysomnographic study, periodic limb movements during sleep (PLMS) decreased significantly in the Mg-supplemented group versus the placebo group (7 PLMS/hr versus 17 PLMS/hr). The overall sleep efficiency in the supplemented cohort significantly improved from 75 to 85% [79].

#### 3.3.9. Magnesium and Smoking Cessation (LOE = B)

Nicotinic cholinergic receptors and NMDA receptors cooperatively contribute to the control of presynaptic dopamine release by nicotine. As mentioned, Mg is a potent inhibitor of the NMDA receptor complex [80]. Mg administration for 4 weeks in heavy adult smokers resulted in a significant decrease in number of cigarettes smoked. Accordingly, Mg may be useful as adjuvant therapy for smoking cessation [81].

#### 3.3.10. Magnesium and Cancer (LOE = A, B)

A decrease in Mg intake reduces intracellular Mg, thus reducing Mg-ATP, in turn increasing cell proliferation by activating Ca channels (TRPM7) which can provide the milieu for development of cancer [82]. A higher ratio of calcium to Mg may increase the risk of postmenopausal breast cancer [83]. Dietary Mg intake appears to be inversely related to a lower risk of developing colorectal adenomas and colorectal cancer [84]. One study showed a 13% reduction in colorectal adenomas for every 100 mg/day increase in Mg intake [84]. Other studies also show a modest 7% risk reduction with 100 mg/day increase in Mg intake [85].

#### 3.3.11. Magnesium and Renal Calculi (LOE = B)

Mg intake with meals is well recognized to bind consumed oxalates in the intestinal tract, thus diminishing oxalate absorption and accrual within the body. As most kidney stones are made up of calcium oxalate, diminution of oxalate content in the body has been found to diminish the risk of stone formation. The use of potassium citrate 1500 mg and Mg citrate 250 mg daily reduced the number of calcium oxalate stones in 64 patients compared to placebo by an impressive 85% over a 3-year period [86]. Further research is required to more adequately study the impact of Mg sufficiency on renal calculi.

#### 3.3.12. Magnesium, Cardiovascular Health, Hypertension, and Sudden Cardiac Death (LOE = A, B)

Magnesium deficiency may affect several different pathophysiological steps involved in the development of arteriosclerosis. Low Mg contributes to vascular calcification, accumulation of connective tissue in the vessel wall, altered lipid exchange between the vessel walls and blood, increased triglycerides, accumulation of oxalate in vessel walls, and reduced cholesterol transport by HDL [87]. Supplementation with oral Mg in elderly diabetic patients (4.5 g/day of Mg pidolate equivalent to 368 mg/day of mg ion) has been shown to improve vascular and endothelial function [88].

Patients with the highest quartile of Mg intake had a reduction of sudden cardiac death by 77% [89, 90]. Oral Mg acts as a natural calcium channel blocker, increases nitric oxide, improves endothelial dysfunction, and induces direct and indirect vasodilation [91]. There is evidence that Mg deficiency may induce resistance to the effects of antihypertensive agents [92]. 500–1000 mg/day of Mg may reduce systolic/diastolic blood pressure as much as 5.6/2.8 mm Hg, although studies vary in the range of reduction [91]. A Cochrane review in 2006 suggested that there was not yet enough information from studies to make conclusive recommendations for the use of Mg in hypertension, despite a small statistical reduction in diastolic blood pressure [93].

#### 3.3.13. Magnesium and Cardiac Arrhythmias (LOE = A, B)

Magnesium has been found to be beneficial in the treatment of digoxin toxicity, torsades de pointes (prolongation of QT interval), and any serious atrial or ventricular arrhythmia, where there is coexistent hypokalemia. A dosage of 2 grams of IV over 10–15 minutes and repeated once if necessary has been found to provide benefit [6].

#### 3.3.14. Magnesium and Atrial Fibrillation (LOE = B)

Magnesium administration has been shown to reduce atrial fibrillation in patients undergoing cardiopulmonary bypass [94] or coronary artery bypass graft surgery [95]. A recent Framingham Heart study with 3530 participants found that low serum Mg was moderately associated with the development of atrial fibrillation [96]. Magnesium depletion also resulted in atrial fibrillation in a number of patients who were deprived of Mg to about 33% of RDA requirements. Atrial fibrillation has been found in some cases to rapidly resolve with repletion of the Mg [97]. Magnesium is also considered a safe and effective treatment in situations of acute atrial fibrillation [98].

#### 3.3.15. Magnesium, Mitral Valve Prolapse, and Congestive Heart Failure (LOE = B, C)

In mitral valve prolapse, Mg supplementation for at least one year has been shown to improve symptoms or result in complete remission of symptoms in a third of individuals [99]. With the use of Mg orotate, patients in severe congestive heart failure have significant improvement in symptoms and survival outcomes as compared to placebo [100].

#### 3.3.16. Magnesium, Chronic Kidney Disease, and Dialysis (LOE-B)

Hypomagnesemia may occur with dialysis, and supplementation with phosphate binders may improve Mg levels that are important to prevent vascular calcification, decrease inflammation, and decrease mortality [101]. Mg supplementation may have favourable effects on intestinal phosphate absorption and vascular calcification in chronic kidney disease [102]. Low Mg levels may also be a risk factor for cardiovascular mortality in patients on maintenance hemodialysis [103].

#### 3.3.17. Lipids and Magnesium (LOE = B)

Both statins and normal Mg levels prevent clotting, reduce inflammation, and prevent atherosclerotic plaques; both approaches have similar pleiotropic effects. Magnesium is a HMG-CoA reductase controller rather than an inhibitor. Statin drugs yield lower LDL-C compared to Mg supplements, but Mg improves all aspects of dyslipidemia by also raising HDL-C and lowering triglycerides [104] without potential adverse effects linked to consumption of statin agents. A significant reduction in total cholesterol and LDL with an increase in HDL has been observed in diabetic patients treated for 12 weeks with 600 mg of Mg [105].

#### 3.3.18. Magnesium, Premenstrual Syndrome, and Hot Flushes in Breast Cancer Therapy (LOE = B)

An RCT using Mg pyrrolidone carboxylic acid (360 mg, 3 times a day) for two cycles resulted in a significantly reduced score on the menstrual distress questionnaire with diminished pain and less mood changes in the supplemented group [106]. In another study, women on tamoxifen or aromatase inhibitors were treated with 400–800 mg of Mg oxide. The hot flush score was reduced by 50.4% and fatigue, sweating, and distress were all significantly reduced with minimal side effects in the treated cohort [107].

#### 3.3.19. Cataract, Glaucoma, and Magnesium (LOE = B)

Senile cataract is the most common cause of bilateral blindness and results from the loss of transparency of the lens. Membrane transport mechanisms utilizing several Mg-dependent ATPases play an important role in maintaining lens homeostasis. Mg supplementation may be of therapeutic value in preventing the onset and progression of cataracts in conditions associated with Mg deficiency [108]. Mg also plays an important role in diminishing the risk of glaucoma by improving ocular blood flow and preventing loss of ganglion cells [109].

#### 3.3.20. Magnesium, Stress, Physical Performance, Aging, and Longevity (LOE = B)

In the presence of Mg deficiency, stress may increase risk of cardiovascular damage, constriction or occlusion of coronary or cerebrovascular arteries, cardiac arrhythmias, and sudden death [110]. Mg infusion may also reduce adrenalin release in autonomic dysfunction [111]. Stress, whether physical stress (including heat, cold, exertion, trauma, or surgery), emotional stress (including excitement, anxiety, or depression), or dyspnea such as that found in asthma, increases the need for Mg [110].

Muscle performance in older individuals (grip strength, lower leg muscle power, knee extension torque, and ankle extension isometric strength) positively correlated with higher SMC [112]. Oral Mg supplementation (Mg oxide 300 mg/day for 12 weeks) in a randomized controlled trial (*n* = 139) resulted in increased physical performance in elderly women as assessed by the Short Physical Performance Battery (SPPB). These findings were more pronounced in women with a Mg dietary intake below the RDA [113].

Space flight and microgravity environments result in accelerated aging with decline of cardiovascular function (increased oxidative stress, insulin resistance, inflammation, and mitochondrial damage). Mg protects against these adverse effects and the shortening of telomeres seen with lower Mg and a reduction of life expectancy [114]. Magnesium deficiency in aging may explain many age-related diseases via a common pathophysiological pathway.

#### 3.3.21. Magnesium and Neurologic Conditions (LOE-B)

Mg deficiency is common in children with attention deficit hyperactivity disorder [115] and supplementation has shown significant improvement in indices of attention and hyperactivity [116, 117]. Mg deficient diets may also contribute to higher fatigue scores in multiple sclerosis patients [118] and Mg supplementation may be a useful adjuvant in improving memory in patients with dementia [119]. Serum ionized magnesium levels correlate with the level of cognitive function, while total serum magnesium levels do not [21]. As emerging evidence suggests that several psychopathological states, including schizophrenia, may be associated with metabolic changes involving Mg, supplementation with this required element is being investigated as an adjuvant therapy for some mental health conditions [120]. Low Mg levels along with high aluminum levels are consistently seen in Parkinson's disease and are believed to contribute to the pathogenesis of this disease [121].

#### 3.3.22. Magnesium and Skin Conditions (LOE-B)

Serum Mg and erythrocyte zinc levels have been shown to be lower in children with atopic dermatitis than in controls [122]. Furthermore, Mg salts are known to enhance skin hydration, dermal permeability, and barrier repair and to facilitate epidermal proliferation and differentiation, thus reducing inflammation [123, 124]. A double-blind controlled trial using a cream containing Mg along with ceramides (a family of waxy lipid molecules found in high concentrations within the cell membrane) to treat mild-to-moderate atopic dermatitis was found to be superior to hydrocortisone creams [125]. Accordingly, there may be beneficial uses of Mg for assorted skin conditions.

### 3.4. Magnesium Sources, Drug Interactions, Toxicity, and Treatment

As well as Mg supplementation, it is important to be aware of several dietary factors that can influence Mg indices. Food processing as occurs with many foods found in the everyday diet of people in the developed world, such as processed white flour or rice, has been found to lower Mg by up to 300–400%. Phytic acid, a natural chelator found in certain foods such as nuts, seeds, and grains including hemp seed [126], can diminish the absorption of required elements including Ca, Fe, Mg, and Zn. Glyphosate, the most common pesticide agent used in the world today, has been found to chelate minerals [29]. On the other hand, ingestion of traditional plants and foodstuffs can greatly improve Mg status. Acidification by using sourdough, for example, improves Mg bioavailability [127].

Accordingly, food selection can largely impact the status of Mg and thus health outcomes. Mg repletion is of particular importance in seniors as absorption of Mg appears to be inversely related to chronological age and may be reduced by more than 30% in the elderly [86]. Sources of Mg, bioavailability of supplements, and potential therapeutic uses are listed in Tables 6 and 7.

Transdermal use of Mg is being used in many individuals who have difficulty tolerating oral Mg [128]. There is insufficient published research in the medical literature on the use and absorption of these agents but various lotions, creams, sprays, oils, compresses, and bath products are in common use to purportedly compensate for insufficient oral intake in some individuals. Other delivery mechanisms being explored include nebulized Mg and eye and ear drops, as well as vaginal Mg douches.

Mg interacts with numerous medications: some drugs reduce while others increase levels of Mg. Some medications prevent absorption of Mg (such as protein pump inhibitors) and need to be stopped in order to restore Mg levels, while other pharmaceutical agents such as selected psychoactive agents seem to elevate Mg levels. Medications that interact with Mg are listed in Table 8.

The Institute of Medicine (IOM) has set the upper tolerable limit of Mg supplementation with no side effects at 350 mg/day (no risk of gastrointestinal side effects in almost all individuals). Although gastrointestinal side effects are often the initial indicator that Mg levels may be excessive, such side effects may vary depending on the type of Mg salt ingested. It is important to note that individuals with renal impairment are at higher risk for adverse effects [15]. Accordingly, it is important to be mindful of potential toxicity. Signs and symptoms of toxicity and the recommended treatment are listed in Table 9.

## 4. Conclusion

The concept of chronic latent Mg depletion is relatively new, yet deficiency of this required biochemical element has been shown to be an often unrecognized and widespread reality in the modern world. Furthermore, insufficient Mg has been linked to a spectrum of clinical afflictions, a not surprising finding considering the required role of the electrolyte in hundreds of essential biochemical reactions. Mg supplementation in appropriate clinical situations appears to be immensely useful in the management of a number of potentially serious and chronic medical conditions.

With the Institute of Medicine (IOM) setting an upper tolerable limit for supplementation without side effects at the considerable dosage of 350 mg/day, there is a substantial margin of safety; intoxication with Mg is rare. Higher levels can be used when indicated and excess Mg will usually result in bowel intolerance such as diarrhea as the first sign of excess. As the half-life of Mg is 42 days, ongoing correction of this deficiency may require long-term supplementation for those not able to secure sufficiency from diet.

There is good evidence for the use of supplemental Mg in preeclampsia/eclampsia, various cardiac arrhythmias, migraine headache, metabolic syndrome, diabetes and diabetic complications, premenstrual syndrome, hyperlipidemia, and asthma. Magnesium should also be considered as an adjunct for depression, attention deficit disorder, prevention of renal calculi, prevention of cataracts, smoking cessation, and a number of other conditions as outlined above. With the alarming increase in diabetes in the general population, it would be prudent to optimize Mg intake for prevention and also for use as an adjuvant agent in the management of this common disorder.

It is evident from the scientific and medical literature that repletion and maintenance of Mg sufficiency can have a profound impact on many common clinical conditions routinely seen and managed by health practitioners. The lack of practical training in clinical nutritional biochemistry within medical education [5] remains an ongoing issue of enormous importance and perhaps represents a significant determinant of the widespread problem of Mg insufficiency. Applied education on the assessment and practical management of nutrient compromise, including Mg deficiency, needs to be provided in medical education in order to overcome and diminish the risk of acquiring chronic disease states associated with biochemical inadequacy.

## Figures and Tables

**Table 1:** Magnesium physiology [13].

Magnesium is involved in over 300 enzyme systems necessary for:
(i) Protein synthesis
(ii) Muscle contraction
(iii) Nerve function
(iv) Blood glucose control
(v) Hormone receptor binding
(vi) Blood pressure regulation
(vii) Cardiac excitability
(viii) Transmembrane ion flux
(ix) Gating of calcium channels

Magnesium is involved in energy production:
(i) Crucial for ATP metabolism (adenylate cyclase)
(ii) Oxidative phosphorylation
(iii) Glycolysis

Nucleic acid synthesis:
(i) Synthesis of RNA and DNA [6]

**Table 2:** Measurement of magnesium levels [6, 7].

(i) Total serum magnesium concentration
Filterable = 33% bound to proteins
25% bound to albumin and 8% to globulin
Unfilterable = 66% of which
92% is free and
8% is complexed to phosphate, citrate, or other compounds
[14]
(ii) Serum ionized Mg concentrate
(iii) Total and free red blood cell Mg concentration
(iv) Tissue Mg from muscle and bone
(v) NMR for free Mg in tissues (research test)
(vi) Magnesium retention test (research test)

**Table 3:** Recommended dietary allowances (RDA) for magnesium in mg [15].

Age	Male	Female	Pregnancy	Lactation
Birth: 6 months	30	30		
7–12 months	75	75		
1–3 years	80	30		
4–8 years	130	130		
9–13 years	240	240		
14–18 years	410	360	400	360
19–30 years	400	310	350	310
31–50	420	310	360	320
51+	420	320		

**Table 4:** Clinical symptoms and signs of magnesium deficiency.

(i) Clinical signs are usually totally absent (chronic latent intracellular deficit)
(ii) Neuromuscular: weakness; tremor; muscle fasciculation; dysphagia; positive Chvostek's sign (facial twitching as a reaction to facial nerve tapping); positive Trousseau's sign (application of a pressure cuff to transiently occlude the brachial artery resulting in spasm of muscles of the hand and forearm)
(iii) Cardiac: arrhythmias and ECG changes
(iv) Central nervous system: depression, agitation, psychosis, nystagmus, and seizures

**Table 5:** Etiology of magnesium deficiency [6].

(i) Reduced dietary intake (processed foods)
(ii) Reduced gastrointestinal absorption (vitamin D deficiency)
(iii) Loss of magnesium from the gastrointestinal tract
Diarrhea and vomiting (acute)
(a) Chronic diarrhea and fat malabsorption:
(1) Celiac disease (all patients with this are deficient) [16]
(2) Regional enteritis
(3) Crohn's disease may require as much as 700 mg/day of magnesium [17]
(4) Resection or small intestine bypass
(5) Laxative use
(iv) Increased renal loss (on average 30% of dietary intake is lost in urine) [18]
(a) Diabetes mellitus/insulin resistance
(1) Due to renal excretion as a result of higher glucose concentrations in the kidney resulting in increased urine output
(b) Alcoholism
(1) Due to decreased intake, gastrointestinal problems, vomiting, phosphate depletion, renal dysfunction, vitamin D deficiency
[19]
(c) Medication induced (see Table 8)
(v) Excessive sweating
(a) On average 10–15% of total output of magnesium may be recovered in sweat
(vi) Increased requirements (pregnancy and growth)
(vii) Older adults: due to lower magnesium intake, decreased absorption, increased renal excretion

**Table 6:** Selected magnesium sources, supplement bioavailability/pharmacodynamics properties, and therapeutic uses.

*Magnesium salts*
(1) Magnesium oxide: bioavailability is poor; effervescent magnesium oxide is better absorbed (8%) than tablets (4%)
(2) Magnesium hydroxide: poorly absorbed; used as an antacid and a cathartic
(3) Magnesium chloride, lactate, and aspartate: have higher and similar bioavailability
(4) Magnesium citrate: significantly better absorbed than oxide since it is more soluble
(5) Magnesium citrate along with potassium citrate are used in nephrolithiasis (prevents absorption of oxalates and citrate chelates oxalate
and urate to prevent stone formation)
(6) Magnesium orotate may be useful in heart failure
(7) Magnesium as a salicylate: is used in rheumatoid arthritis
(8) Magnesium mandelate: is used as urinary antiseptic
(9) Magnesium glycinate or taurinate: has been used in depression
(10) Magnesium from magnesium-rich mineral water: 59% absorption

**Table 7:** Common food sources of magnesium (in mg per serving or 100 gm).

*Seeds*	*Mg/serving*
(i) Hemp seeds (100 gm)	700
(ii) Pumpkin seeds (100 gm)	535
(iii) Flax seeds (100 gm)	392
(iv) Brazil nuts (100 gm)	376

*Carbohydrates*	*Mg/serving*
(i) Whole wheat bread (2 slices)	46
(ii) Baked potato (3.5 ounces)	43
(iii) Rice, brown rice (1/2 cup)	42
(iv) Kidney beans (1/2 cup)	35
(v) White rice (1/2 cup)	10

*Greens*	
(i) Boiled spinach (1/2 cup)	78
(ii) Avocado (cubed 1 cup)	44
(iii) Broccoli (chopped, cooked 1/2 cup)	12

*Others*	
(i) Yogurt (low-fat 8 ounces)	42
(ii) Milk (8 ounces)	24–27
(iii) Farmed Atlantic Salmon (3 ounces)	26
(iv) Cooked halibut (3 ounces)	24
(v) Roasted chicken breast (3 ounces)	22
(vi) Chopped and cooked beef (3 ounces)	20
(vii) Apple	9
(viii) Raw carrots (one medium)	7
(ix) Raisins (1/2 cup)	23

Source: the US Department of Agriculture's (USDA) Nutrient Database website.

**Table 8:** Magnesium and drug interactions.

Medications that reduce magnesium levels:
(i) *H2 blockers*: for example, cimetidine and nizatidine
(ii) *Proton pump inhibitors*: for example, esomeprazole, omeprazole, and pantoprazole (*FDA WARNING*: supplementing magnesium will not correct deficiency; you must stop the drug)
(iii) *Antacids*: aluminum and magnesium hydroxide and sodium bicarbonate
(iv) *Antibiotics*: for example, amoxicillin, azithromycin, doxycycline, minocycline, levofloxacin, ciprofloxacin, cephalexin,
sulfamethoxazole and trimethoprim, and tetracycline
(v) *Antihistamines*: for example, astemizole and terfenadine
(vi) *Antivirals*: for example, delavirdine, lamivudine, and zidovudine
(vii) *Antiepileptic medications*: phenytoin and phenobarbital
(viii) *Blood pressure drugs*: hydralazine and combination of ACE inhibitors with HCTZ (enalapril and HCTZ)
(ix) *Diuretics*: for example, furosemide, ethacrynic acid, chlorothiazide, chlorthalidone, metolazone, and indapamide
(x) *Cardiac glycoside*: digoxin
(xi) *Cardiac drugs*: sotalol, amiodarone, bretylium, and quinidine
(xii) *CNS stimulants*: methylphenidate
(xiii) *Cholesterol agents*: cholestyramine and colestipol
(xiv) *Corticosteroids*: betamethasone, dexamethasone, hydrocortisone, prednisone, and triamcinolone
(xv) *Inhaled corticosteroids*: fluticasone, flunisolide, and triamcinolone
(xvi) *Estrogens*: DES, estradiol, estring, and estrogen-containing drugs: HRT and BCP
(xvii) *Immunosuppressants*: cyclosporine and tacrolimus
(xviii) *Nonsteroidal aromatase inhibitors for breast cancer*: anastrozole
(xix) *Osteoporosis*: raloxifene
(a) On the other hand, magnesium decreases bisphosphonate absorption
(xx) *SERMs (selective estrogen receptor modulators)*: raloxifene, tamoxifen, and toremifene
(xxi) *Sulfonamides*: antibiotics and some diabetic medications
(xxii) *Nutraceuticals*: for example, high-dose calcium, high-dose vitamin D, and caffeine

Medications that may increase serum magnesium:
(i) *Lithium carbonate*
(ii) *Antidepressants*: for example, sertraline and amitriptyline
(iii) *Potassium sparing diuretics*: amiloride and spironolactone reduce magnesium excretion

**Table 9:** Signs and symptoms of magnesium toxicity and clinical management.

Laxative effect, diarrhea
Fall in blood pressure with dizziness to severe hypotension
Muscle weakness (and depressed deep tendon reflexes)
Severe back pain and pelvic pain
Confusion and loss of consciousness
Difficulty breathing to respiratory arrest
Cardiac arrhythmias to cardiac arrest
Other effects: lethargy, confusion, deterioration of kidney function

Treatment:
Mild magnesium overdose:
(i) Discontinue over the counter magnesium-containing laxatives, antacids, or magnesium supplements and rule out renal impairment
Severe magnesium overdose (>1.1 mmol/l)
(i) Artificial respiratory support may be needed
(ii) Intravenous fluids (saline diuresis) and furosemide
(iii) IV calcium gluconate or calcium chloride (10% solution 500–1000 mg IV)
(iv) Renal dialysis
